# Re: Can Concomitant Bladder Neck Incision and Primary Valve Ablation Reduce Early Re-admission Rate and Secondary Intervention?

**DOI:** 10.1590/S1677-5538.IBJU.2021.0383.1

**Published:** 2022-04-10

**Authors:** Ubirajara O Barroso

**Affiliations:** 1 Universidade Federal da Bahia Escola Bahiana de Medicina Salvador BA Brasil Disciplina de Urologia, Universidade Federal da Bahia e Escola Bahiana de Medicina Salvador, BA, Brasil

*To the editor,*

Is it necessary to perform a bladder neck incision (BNI) in patients with a posterior urethral valve (PUV)? In this edition of the International Brazilian Journal of Urology, Abdelhalim et al. ( 1 ) addresses this question and thereby adds data to the literature concerning this topic.

Some studies have demonstrated that PUV ablation + BNI can be more effective than ablation alone ( 2 , 3 ). In a randomized control trial by Singh et al, PUV ablation + BNI was more effective than PUV ablation alone in terms of achieving maximal urinary flow and the reduction of post-void residual but was similar not only regarding other urodynamic parameters such as compliance, bladder capacity, and detrusor overactivity but also in the resolution of vesicoureteral reflux ( 2 ). Kajbafzadeh et al., in a study regarded by the authors as prospective, found a lower rate of reintervention, less use of anticholinergics, and less need for CIC in the group with PUV ablation + BNI compared with the PUV ablation alone group. ( 2 ) The study by Kajbafzadeh et al, however, does not make clear what were the selection criteria for one treatment or the other.

The major limitation of the two studies is the fact that the group undergoing PUV ablation alone did not systematically use alpha1-blockers. In the study by Singh et al, only about 20% of patients used alpha-blockers in the control group (PVU ablation alone) ( 2 ), while in the study by Kajbafzadeh et al et al this information is not given, though it seems clear that the use of alpha-blockers was not part of the study protocol ( 3 ).

On the other hand, Abdelhalim et al. ( 1 ) have shown that there is no need to perform BNI together with PUV ablation, since patients who underwent BNI had the same reoperation rate as those who underwent PUV alone. Other studies have not shown a significant difference in urodynamic improvement in the BNI + PUV ablation group ( 4 ).

The main reason for not performing BNI together with PUV ablation is the lack of studies that have been completed that compare this procedure with the use of alpha 1 blockers of the bladder neck. Since there is no current study that shows the superiority of BNI over alpha-blockers, this procedure should not be used routinely. As an example, Androulakakis et al. reported on 5 patients with underactive bladder secondary to PUV being treated successfully, one by BNI and 4 with alpha-blockers, which demonstrates the efficacy of this medication ( 5 ). Others have reported satisfactory results with alpha-blockers in patients with VUP ( 6 ).

We routinely use alpha-blockers such as doxazosin, 1mg, even before a patient’s first year of life; however, this does not mean that BNI is contraindicated. Some patients will not respond well to this medication and will exhibit high post-voiding residue, recurrent urinary tract infection, or worsening renal function. These are the cases in which we opted for BNI at the same time as we performed a cystoscopy to review a possible valve persistence.

Patients with a posterior urethral valve most often have a hypertrophied bladder neck. The justification for not performing BNI, which concerns the risk of retrograde ejaculation or urinary incontinence, does not seem to be supported by medium-term studies ( 5 , 6 ). BNI is a well-tolerated procedure without any significant increased risk of bleeding, increased postoperative pain, longer hospital stays, or significant cost increases.

In conclusion, based upon the interpretation of the literature, PUV ablation can be performed alone without additional procedure; however, the valve bladder must be aggressively treated with alpha-blockers and oxybutynin most of the time. In the future, BNI may be necessary in cases of unfavorable evolution.

The Author
